# A Cross-Sectional Study on the Dietary Pattern Impact on Cardiovascular Disease Biomarkers in Malaysia

**DOI:** 10.1038/s41598-019-49911-6

**Published:** 2019-09-20

**Authors:** Tilakavati Karupaiah, Khun-Aik Chuah, Karuthan Chinna, Peter Pressman, Roger A. Clemens, A. Wallace Hayes, Kalyana Sundram

**Affiliations:** 1 School of BioSciences, Faculty of Health & Medical Sciences, Taylor’s University, Selangor, Malaysia; 2 School of Healthcare Sciences, Faculty of Health Sciences, National University of Malaysia, Selangor, Malaysia; 3 School of Medicine, Faculty of Health & Medical Sciences, Taylor’s University, Selangor, Malaysia; 4 Polyscience Consulting & Director of Nutrition and Public Health, The Daedalus Foundation, San Clemente, CA USA; 5 Pharmacology & Pharmaceutical Sciences, USC School of Pharmacy, International Center for Regulatory Science, Los Angeles, CA, USA; 6 College of Public Health, University of South Florida, Tampa, FL USA; 7 Malaysian Palm Oil Council, Wisma Sawit, Jalan Perbandaran, Kelana Jaya, Selangor, Malaysia

**Keywords:** Lipidomics, Lipoproteins

## Abstract

We conducted this cross-sectional population study with a healthy multi-ethnic urban population (n = 577) in Malaysia, combining nutritional assessments with cardiometabolic biomarkers defined by lipid, atherogenic lipoproteins, inflammation and insulin resistance. We found diametrically opposing associations of carbohydrate (246·6 ± 57·7 g, 54·3 ± 6·5%-TEI) and fat (total = 64·5 ± 19·8 g, 31·6 ± 5·5%-TEI; saturated fat = 14·1 ± 2·7%-TEI) intakes as regards waist circumference, HDL-C, blood pressure, glucose, insulin and HOMA2-IR as well as the large-LDL and large-HDL lipoprotein particles. Diets were then differentiated into either low fat (LF, <30% TEI or <50 g) or high fat (HF, >35% TEI or >70 g) and low carbohydrate (LC, <210 g) or high carbohydrate (HC, >285 g) which yielded LFLC, LFHC, HFLC and HFHC groupings. Cardiometabolic biomarkers were not significantly different (*P* > 0.05) between LFLC and HFLC groups. LFLC had significantly higher large-LDL particle concentrations compared to HFHC. HOMA-IR2 was significantly higher with HFHC (1·91 ± 1·85, *P* < 0·001) versus other fat-carbohydrate combinations (LFLC = 1·34 ± 1·07, HFLC = 1·41 ± 1·07; LFHC = 1·31 ± 0·93). After co-variate adjustment, odds of having HOMA2-IR >1.7 in the HFHC group was 2.43 (95% CI: 1·03, 5·72) times more compared to LFLC while odds of having large-LDL <450 nmol/L in the HFHC group was 1.91 (95% CI: 1·06, 3·44) more compared to latter group. Our data suggests that a HFHC dietary combination in Malaysian adults is associated with significant impact on lipoprotein particles and insulin resistance.

## Introduction

The rising global burden of diet-related non-communicable diseases (NCDs) impacts health equity and economic productivity in developing countries, as mortality from NCDs occur during the middle life years^1–3^. The mortality toll from NCDs largely contributed by cardiovascular disease (CVD) is 73% in Malaysia for the last decade, alongside a rapidly urbanizing society with a growing middle class^4,5^. Food availability has increased, and for almost 70% of adults, fruit and vegetable consumption has decreased coupled with increased caloric consumption from fats and excess sugar and sedentary lifestyle^6^. Despite these developments, there is scarce Malaysian data linking local dietary patterns to NCD risk.

Dramatic changes in food consumption patterns may also reflect or incorporate major modifiable risk factors for NCDs. The public health risk burden has compelled the *WHO Global NCD Action Plan 2013–2020* to advocate dietary changes, especially moving populations’ dietary fat consumption towards more unsaturated fats through reformulation, labelling and fiscal and agricultural policies^7,8^. These food-based dietary guidelines target unsatisfactory dietary intakes associated with NCDs, including excess intake of unhealthy sugars and sugary beverages, fats, and salt, coupled with poor intake of fruits and vegetables^6^. Current dietary guidelines of the United States and United Kingdom also opt to adopt these strategies to lower the risk of CVD^9,10^. Despite these recommendations, the evidence base for dietary guidelines in general has been challenged, in relation to their potential CVD risk reduction efficacy, especially those recommendations targeting saturated fats, as the global pandemic of NCDs continues unabated with CVD as the largest contributor to mortality^4,11,12^. In particular, the recently published 18-country PURE prospective cohort study including Malaysia, highlights high carbohydrate intake rather than total fat or saturated fat were related to higher mortality risk from CVDs^13^.

Given this gap in the knowledge between dietary consumption patterns and CVD risk in the Malaysian population, we designed and undertook the *Malaysia Lipid Study* (MLS) to address key questions related to dietary macronutrients’ consumption and their association with CVD and diabetes. This cross-sectional study goes beyond the traditional metabolic syndrome (MetS) parameters, to elucidate other overlapping risk factors such as insulin resistance and vasculopathy assessed through the lipoprotein subclasses of which small dense LDL particles have been shown to be a better predictor for CVD than plasma LDL-C^14–16^.

## Methods

### Study population

The Malaysia Lipid Study (MLS) is a cross-sectional study investigating dietary practices and metabolic outcomes in an urban, mixed-racial population of healthy free-living adults. Malays, Chinese and Indians are among the main ethnic groups in Malaysia, and together form approximately 85% of the total population^17^. This study was conducted according to the guidelines laid down in the Declaration of Helsinki and all procedures involving human subjects were approved by the Medical Ethics Committee of the National University of Malaysia (UKM 1.5.3.5/138/NN-047-2012), and the protocol was registered with the National Medical Research Register, Malaysia (nmrr@nmrr.gov.my, ID: NMRR-15-33-23993). Written, informed consent was obtained from study participants prior to study enrolment.

Participant screening and subject recruitment were conducted in the urban centers of Kuala Lumpur and Petaling Jaya and surrounding suburban housing estates. Screening was facilitated through religious, community, parent-teacher associations and employer organizations at 38 community sites, between November 2012 and November 2013. Potential participants arrived at the study centre following a 12-hour overnight fasting in light clothing. Study protocols and procedures were explained to participants by the research team members. All participants underwent a preliminary medical examination performed by a medical doctor before informed consent was signed and enrolment into the study. Eligibility criteria for recruitment included (1) age between 20–65 years old (2) free-living status, and (3) freedom from medical conditions such as diabetes, hypertension, coronary artery disease, stroke, cancer, renal failure or hypothyroidism. Those on cholesterol-lowering medication, adherent to weight loss or muscle building therapies, heavy smokers (>10 cigarettes per day) and alcohol consumption (>2 standard drinks per day) were also excluded. Pregnant, breastfeeding or menopausal women were excluded.

Then participants willing to comply with study protocols, signed the informed consent before they were recruited into the MLS. Participants who did not pass the medical examination or had been diagnosed with a medical condition by the MLS physicians, were excluded from this study. Of 2,790 participants attending the MLS screening sessions, we initially shortlisted 598 subjects with adequate ethnic representation.

### Clinical assessment

Shortlisted subjects underwent a medical screening conducted by physicians and dietitians. The screening included collection of fasted blood samples as well as measurements for weight, height, waist circumference, body composition and blood pressure.

### Lifestyle information

We also collected detailed information on dietary history through self-recorded food records for three days (two different weekdays and one weekend) prior to subjects’ screening at the study center. The collection of dietary data followed the 24-hour dietary recall methodology cited by NHANES^18^. These records were verified to ensure completeness of information. Nutrient intake was analyzed using Nutritionist-Pro software (First-databank, Chicago, IL, USA), which crosslinks to the Malaysian, Singapore and USDA Food Composition databases^19^. Information on any dietary supplement use and detailed history of dietary fat and cholesterol intake were also collected during the dietary interview. Additionally, physical activity level (PAL) of subjects was assessed through the long form of the International Physical Activity Questionnaire, and calculated as metabolic equivalent (MET) scores^20^.

### Blood collection and analyses

Twenty-ml blood sample was drawn from fasting subjects, from which 16 ml were collected into Vacutainer® tubes (Becton Dickinson Vacutainer, NJ, USA) for analysis of lipids and fatty acids, while 4 ml blood were collected into lithium heparin tubes for insulin analysis. Prepared plasmas were immediately transferred into 1·5 ml storage tubes, snap-frozen in liquid nitrogen and stored at −80 °C until further analysis. To reduce analytical intra-variations, all plasma samples were analyzed in a single batch.

### Analyses of lipids and lipoproteins

Plasma total cholesterol (TC), HDL-C and triglycerides (TG) were analyzed by enzymatic procedures, using a Cobas 6000 Chemistry Autoanalyzer (Roche Analytic Instruments Inc, Nutley, NJ) supplied with reagents, calibrators and controls by the manufacturer (Roche Diagnostics Corporation, Indianapolis, IN)^21^. LDL-C was calculated using the Friedewald equation, while VLDL-C was calculated from TG values multiplied by a factor of 0·46^21,22^.

### Determination of glucose, insulin and HOMA2-IR

Plasma glucose was assayed using an automated *in vitro* step enzymatic procedure diagnostic reagent kit, Glucose HK Liquid (GLUCL 0–992) (Roche Diagnostics Corporation, Indianapolis, IN). Insulin was analyzed using heparinized plasma by automated electrochemiluminescence (ROCHE Elecsys 2010 Modular E170 system, Roche Diagnostics, Switzerland) involving a sandwich immunoassay with streptavidin-coated micro particles in two phases of incubation to quantify the insulin in 20 μL of plasma. We used the computer model of Homeostasis Model Assessment (HOMA2) to calculate insulin resistance (IR)^15^. This HOMA2-IR version accounts for variations in hepatic and peripheral glucose resistance and the reduction of peripheral glucose-stimulated glucose uptake. A score higher than 1·7 was considered as at-risk for insulin resistance^15^.

### Determination of high-sensitivity C-reactive protein (hsCRP)

As a marker of inflammation, hsCRP was measured by nephelometric turbidimetric immunoassay by automated analyses^23^.

### NMR based lipoprotein particle (LP) measures

Particle concentrations and particle size profiles for lipoprotein subclasses were carried out by automated nuclear magnetic resonance (NMR) spectroscopic assay by an independent laboratory [LipoScience Inc., Raleigh, North Carolina, USA]. EDTA plasma samples stored at −80 C were shipped in dry ice to the certified laboratory [CLIA ID No. 34D0952253] as per service provider instructions.

### Statistical analysis

**Figure 1:**
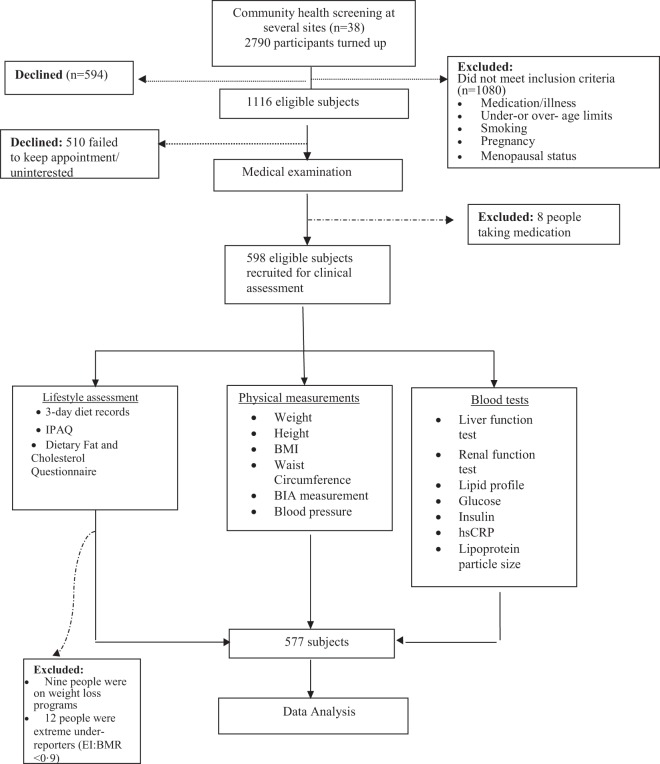
Study flow from participant screening to subject recruitment to data analysis. Abbreviations: BIA = Bioimpedence analysis; BMI = body mass index; EI:BMR = energy intake: basal metabolic rate; IPAQ = International Physical Activity Questionnaire.

From a total of 598 subjects, 577 subjects were eligible for data analysis after exclusion of extreme diets or under-reporting of diet records (EI:BMR <0·9). Figure 1 presents the study flow from participant screening to subject recruitment and eligibility for final inclusion into the study.

All data were analyzed using SPSS for Windows Version 20 (IBM, Chicago, IL, USA). Qualitative variables were described as frequency and percentage (%) while the quantitative variables were described as mean ± SD. Dietary data was tested for normality. The physical activity status was classified based on the MET scores.

The associations between plasma biomarkers and individual macronutrient intake were examined by using multivariate analyses using the General Linear Model (GLM). The β-coefficient and the 95% confidence interval (CI) were reported for these analyses. The associations were further examined by adjusting for age, BMI, PAL, sex and total energy intake (TEI). The dietary intake of the MLS population was further evaluated for low fat (<30%-TEI) or high fat (>35%-TEI)-carbohydrate combinations based on their calculated mean energy intakes with reported fat intakes distributed between 25–35%-TEI and carbohydrate intakes distributed between 50–60%-TEI^24^. Comparisons of the total energy and macronutrients intake between fat- carbohydrate combinations were performed using *ANOVA*. Multivariate analyses using GLM were performed to determine the effect of carbohydrate-fat combinations on cardio-biomarker profiles and lipoprotein subclasses, unadjusted and adjusted for age, BMI, PAL and sex. Multiple logistic regression was used to determine associations between dietary intake patterns, biomarkers and lipoprotein particle concentrations, unadjusted and adjusted for age, BMI, PAL and sex. The results were expressed as odds ratio (OR) and 95% CI. Level of significance was set at *P* < 0·05 for all analyses.

## Results

### Population characteristics

This typically urban Malaysian study population of 217 men and 360 women (age: 38·3 ± 11·4 y), reflected an ethnic distribution of 228 Malays (40%), 203 Chinese (35%) and 146 Indians (25%) (Table 1). In this population, 51·6% of subjects reported moderate physical activity whilst 21·7% were sedentary. This population largely belonged to low (49.6%) and medium (32.4%) income households, completed high school (92.7%) and tertiary (59.7%) education, and held white collar jobs (59.4%). No one in this population smoked or consumed alcohol. Prevalence of MetS was 16·3% as per the Harmonized criteria^14^.

**Table 1:** Population demographics.

Characteristics	Overall (n = 577)
Age, *year*s (range)	38·3 ± 11·4 (20–65)
Gender, *n* (%)	
men	217 (38%)
women	360 (62%)
Ethnicity, *n* (%)	
Malay	228 (40%)
Chinese	203 (35%)
Indian	146 (25%)
Income level, *n* (%)	
Low income (<USD 1265.00 per month)	286 (49·6%)
Medium income (USD 1265.00–2668.00 per month)	187 (32·4%)
High income (>USD 2668.00 per month)	104 (18·0%)
The highest education level, *n* (%)	
No formal education	6 (1·0%)
Primary School	36 (6·3%)
High School	188 (32·5%)
Tertiary, college/diploma	131 (22·8%)
Tertiary, degree and above	213 (36·9%)
Others	3 (0·5%)
Occupation, *n* (%)	
white collar	343 (59·4%)
blue collar	80 (13·9%)
housewives	50 (8·7%)
retired/unemployed	15 (2·6%)
Self-employed/businessman	46 (8·0%)
Students	34 (5·9%)
Others	9 (1·6%)
Smoking (yes/no)	0/577
Alcohol consumption (yes/no)	0/577
Physical Activity Status*, *n* (%)	
Sedentary	125 (21·7)
Moderate	298 (51·6)
High	154 (26·7)
BMI (kg/m^2^)	25·0 ± 4·8
Underweight (<18.5 kg/m^2^)	5.00%
Normal weight (18.5–24.9 kg/m^2^)	51.30%
Overweight (25.0–29.9 kg/m^2^)	28.20%
Obese class I (30.0–34.9 kg/m^2^)	11.10%
Obese class II (>35.0 kg/m^2^)	4.30%
Waist circumference (cm)^†^	84·2 ± 13·3
Normal waist circumference (<90 cm for men, <80 cm for women)	53.20%
Larger waist circumference (≥90 cm for men, ≥80 cm for women)	46.80%
% Body fat	33·0 ± 9·92
Normal body fat (≤24% for men, ≤35% for women)	54.40%
High body fat (>24% for men, >35% for women)	45.60%
BP_s_(mmHg)^†^	123 ± 16
Desirable BP_s_(≤130 mmHg)	72.10%
Undesirable BP_c_(>130 mmHg)	27.90%
BP (mmHg)	75 ± 11
Desirable BP (≤85 mmHg)	83.20%
Undesirable BP (>85 mmHg)	16.80%
MetS Prevalence, *n* (%)^†^	94 (16·3)
Mean Daily Nutrient Intake	
Total energy (kcal)	1825 ± 413
CHO (g) (%-TEI)	246·6 ± 57·7 (54·3 ± 6·5)
Fat (g) (%-TEI)	64·5 ± 19·8 (31·6 ± 5·5)
Protein (g) (%-TEI)	63·5 ± 18·7 (13·9 ± 2·5)
SFA (g) (%-TEI)	28·7 ± 8·9 (14·1 ± 2·7)
MUFA (g) (%-TEI)	25·7 ± 8·6 (12·6 ± 2·7)
PUFA (g) (%-TEI)	9·8 ± 4·2 (4·8 ± 1·6)
P/S ratio	0·48 ± 0·14
POL-users/non-POL users, *n* (%)	484 (83·9)/93 (16·1)

Abbreviations: BPdiastolic = diastolic blood pressure; BPsystolic = systolic blood pressure; CHO = carbohydrate; MUFA = monounsaturated fat; PUFA = polyunsaturated fat; SFA = saturated fat;

%-TEI = percentage of total energy intake; P/S = Polyunsaturated: Saturated fatty acids; MetS = Metabolic syndrome; POL = palm olein.

Notes-

All data are expressed as mean ± SD.

^#^Alcohol consumed at least once per month as one unit equivalent; smokers have been excluded from this study;

*Based on IPAQ scoring (Craig *et al*.)^20^.

^†^Based on Harmonized definition (Alberti *et al*.)^14^.

BMI categories based on WHO (1998)^43^. % Body fat cut-offs based on Gallagher *et al*.^44^.

^‡^Comparison between POL and non-POL users was not significantly different for the cardiometabolic profile (*data not shown)*. Also refer Supplementary Table 1: Total energy and macronutrient intakes as per quartiles.

### Dietary status

The mean daily TEI was 1825 ± 413 kcal, with a macronutrient distribution of 246·6 ± 57·7 g carbohydrate (54·3 ± 6·5%-TEI), 64·5 ± 19·8 g fat (31·6 ± 5·5%-TEI) and 63·5 ± 18·7 g protein (13·9 ± 2·5%-TEI). Energy from dietary fatty acid intake was 14·1 ± 2·7%-TEI from SFA, 12·6 ± 2·7%-TEI from MUFA, and 4·8 ± 1·6%-TEI from PUFA. For 83·9% (n = 484) of subjects, liquid palm oil or palm olein (POL) was the habitual cooking oil at home or in foods consumed at local catering outlets. The remaining non-POL users (n = 93) reported either cooking or consuming foods cooked with other types of vegetable oils for at least three meals a week, and this included regular consumption of foods prepared with sunflower (n = 57), Canola (n = 11), corn (n = 10), or olive (n = 10) oils.

### Associations between cardio-biomarkers and individual macronutrients

Associations between cardio-biomarker profiles and individual macronutrient intake (Table 2) were first examined using multivariate analysis for unadjusted (Model 1), and adjusted for confounding factors which included age, BMI, PAL, sex and TEI (Model 2).

**Table 2:** Cardio-biomarkers and association with dietary nutrient intake.

	Mean ± SD		CHO	*P*-value	Fat	*P*-value	Protein	*P*-value
			β (95%CI)		β (95%CI)		β (95%CI)	
TC (mmol/L)	5.12 ± 0.90	Model 1	0.000 (−0.001, 0.001)	0.984	−0.001 (−0.005, 0.002)	0.460	0.001 (−0.003, 0.005)	0.770
		Model 2	−0.001 (−0.004, 0.001)	0.250	0.003 (−0.003, 0.01)	0.309	0.006 (0.000, 0.012)	0.050
TG (mmol/L)	1.17 ± 0.61	Model 1	0.002 (0.001, 0.003)	<0.001	0.001 (−0.001, 0.004)	0.262	0.002 (0.000, 0.005)	0.095
		Model 2	0.001 (0.000, 0.003)	0.099	−0.003 (−0.007, 0.001)	0.141	−0.002 (−0.006, 0.002)	0.267
VLDL-C (mmol/L)	0.54 ± 0.28	Model 1	0.001 (0.000, 0.001)	<0.001	0.001 (0.000, 0.002)	0.262	0.001 (0.000, 0.002)	0.095
		Model 2	0.001 (0.000, 0.001)	0.099	−0.001 (−0.003, 0.000)	0.141	−0.001 (−0.003, 0.001)	0.267
LDL-C (mmol/L)	3.11 ± 0.86	Model 1	0.000 (−0.001, 0.002)	0.461	0.000 (−0.004, 0.003)	0.855	0.001 (−0.002, 0.005)	0.486
		Model 2	−0.001 (−0.003, 0.001)	0.415	0.002 (−0.004, 0.009)	0.416	0.004 (−0.001, 0.01)	0.144
HDL-C (mmol/L)	1.47 ± 0.39	Model 1	−0.001 (−0.002, −0.001)	<0.001	−0.002 (−0.003, 0.000)	0.036	−0.002 (−0.004, 0.000)	0.042
		Model 2	−0.001 (−0.002, 0.000)	0.028	0.002 (0.000, 0.005)	0.082	0.003 (0.000, 0.005)	0.020
TC:HDL-C	3.72 ± 1.18	Model 1	0.003 (0.002, 0.005)	<0.001	0.004 (−0.001, 0.009)	0.122	0.005 (0.000, 0.010)	0.063
		Model 2	0.001 (−0.001, 0.004)	0.331	−0.002 (−0.009, 0.005)	0.586	−0.003 (−0.01, 0.004)	0.342
LDL:HDL-C	2.30 ± 0.95	Model 1	0.002 (0.001, 0.003)	0.002	0.003 (−0.001, 0.007)	0.186	0.003 (−0.001, 0.008)	0.110
		Model 2	0.001 (−0.002, 0.003)	0.626	0.000 (−0.006, 0.006)	0.923	−0.002 (−0.007, 0.004)	0.567
TG:HDL-C	0.92 ± 0.73	Model 1	0.002 (0.001, 0.003)	<0.001	0.003 (0.000, 0.006)	0.094	0.003 (0.000, 0.006)	0.048
		Model 2	0.002 (0.000, 0.003)	0.064	−0.004 (−0.009, 0.001)	0.128	−0.004 (−0.008, 0.001)	0.121
GLU (mmol/L)	5.21 ± 1.08	Model 1	0.001 (−0.001, 0.002)	0.336	0.005 (0.000, 0.009)	0.044	0.001 (−0.003, 0.006)	0.597
		Model 2	−0.003 (−0.006, −0.001)	0.014	0.011 (0.004, 0.019)	0.004	−0.003 (−0.01, 0.005)	0.470
Insulin (mmol/L)	6.36 ± 4.77	Model 1	0.014 (0.008, 0.021)	<0.001	0.044 (0.025, 0.064)	<0.001	0.035 (0.014, 0.056)	0.001
		Model 2	−0.003 (−0.014, 0.008)	0.598	0.017 (−0.014, 0.047)	0.282	−0.016 (−0.044, 0.013)	0.284
HOMA2-IR	0.86 ± 0.60	Model 1	0.002 (0.001, 0.003)	<0.001	0.006 (0.003, 0.008)	<0.001	0.004 (0.002, 0.007)	0.001
		Model 2	0.000 (−0.002, 0.001)	0.530	0.003 (−0.001, 0.006)	0.189	−0.002 (−0.006, 0.001)	0.207
SBP (mmHg)	123 ± 16	Model 1	0.064 (0.042, 0.086)	<0.001	0.045 (−0.022, 0.111)	0.187	0.101 (0.031, 0.171)	0.005
		Model 2	0.035 (−0.003, 0.073)	0.074	−0.114 (−0.217, −0.011)	0.029	0.008 (−0.089, 0.106)	0.866
DBP (mmHg)	75 ± 11	Model 1	0.039 (0.024, 0.055)	<0.001	0.038 (−0.007, 0.082)	0.099	0.064 (0.017, 0.111)	0.008
		Model 2	0.023 (−0.003, 0.05)	0.083	−0.067 (−0.138, 0.004)	0.066	−0.01 (−0.078, 0.057)	0.761
Waist Circumference (cm)	84.2 ± 13.3	Model 1	0.052 (0.034, 0.071)	<0.001	0.076 (0.022, 0.131)	0.006	0.068 (0.01, 0.126)	0.022
		Model 2	0.024 (0.006, 0.043)	0.008	−0.027 (−0.076, 0.022)	0.284	−0.088 (−0.134, −0.042)	<0.001
hsCRP (mg/L)	3·02 ± 4·68	Model 1	0·001 (−0·006, 0·008)	0·798	−0·014 (−0·033, 0·006)	0·167	−0·016 (−0·036, 0·005)	0·136
		Model 2	0·008 (−0·004, 0·020)	0·190	−0·013 (−0·046, 0·020)	0·427	−0·017 (−0·048, 0·014)	0·278

Abbreviations: BMI = body mass index; BPdiastolic = diastolic blood pressure; BPsystolic = systolic blood pressure; GLM = general linear model; HDL-C = high density lipoprotein cholesterol; HOMA2-IR = homeostatic model assessment of insulin resistance; LDL-C = low density lipoprotein cholesterol; PAL = physical activity level; TC = total cholesterol; TG = triglyceride; VLDL-C = very low density lipoprotein cholesterol.

Note- Carbohydrate, fat and protein intakes were dependent variables with all cardio-biomarkers as independent variables. Model 1: Multivariate analysis (GLM), unadjusted.

Model 2: Multivariate analysis (GLM), adjusted for age, BMI, PAL, sex and total energy intake. Also refer Supplementary Table 2: Cardio-biomarkers profile as per quartiles of total energy and macronutrients intake.

Waist circumference (WC) of subjects was significantly and positively associated with carbohydrate (β = 0·052, *P* < 0·001), fat (β = 0·076, *P* = 0·006) and protein (β = 0·068, *P* = 0·022) intakes in Model 1. With adjustment (Model 2) WC association remained positive with carbohydrate (β = 0·024, *P* = 0·008), became negative with protein (β = −0·088, *P* < 0·001), and neutral with fat (β = −0·027, *P* = 0·284).

The inflammatory marker hsCRP, was not significantly associated with any dietary macronutrient. TC was not associated with any nutrient in crude analysis but showed a weak positive association with protein (β = 0.006, 95% CI: 0.000, 0.012, *p* = 0.050) in adjusted analysis.

Associations of TG (β = 0·002, *P* < 0·001), VLDL-C (β = 0·001, *P* < 0·001), TC:HDL-C (β = 0·003, *P* < 0·001), TG:HDL-C (β = 0·002, *P* < 0·001) were strongly positive and HDL-C (β = *−*0·001*, P* < 0·001) negative only with carbohydrate consumption in Model 1. With co-variate adjustment, the negative association with HDL-C remained with carbohydrate intake (β = −0·001, 95% CI: −0.002, 0.000, *p* = 0·028) but a positive association emerged with protein (β = 0.003, 95% CI: 0.000, 0.005, *p* = 0.020).

Insulin (β = 0·014, *P* < 0·001) and HOMA2-IR (β = 0·002, *P* < 0·001) showed positive associations with carbohydrate and with protein (insulin, β = 0·035, *P* = 0·001 and HOMA2-IR, β = 0·004, *P* = 0·001) intakes in Model 1 but these associations disappeared with co-variate adjustment. Instead glucose associations with macronutrients not discerned with crude analysis, became positively associated with fat (β = 0·011, 95% CI: 0.004, 0.019, *p* = 0·004) and negatively with carbohydrate (β = −0·003, 95% CI: −0.006, −0.001, *p* = 0·014) in Model 2.

In crude analysis, blood pressure was positively associated with both carbohydrate (SBP, β = 0·064, *P* < 0·001 and DBP, β = 0·039, *P* < 0·001) and protein (SBP, β = 0·101, *P* = 0·005 and DBP, β = 0·064, *P* = 0·008) intakes. In adjusted analyses only SBP was observed to be negatively associated with fat (β = −0·114, 95% CI: −0.217, −0.011, *p* = 0·029) intake.

### Associations between lipoprotein particles and individual macronutrients

Associations between various lipoprotein particle numbers and size with individual macronutrients are shown in Table 3. After adjusting for co-variates, only large-LDL, total-HDL and large-HDL particle concentrations and LDL particle size associated with the macronutrients. Large-LDL concentrations were negatively associated with carbohydrate (β = −0·809, 95% CI: −1.396, −0.221, *p* = 0·007) and positively with protein (β = 3·111, 95% CI: 1.614, 4.608, *p* < 0·001). Whereas large-HDL concentrations were negatively associated with carbohydrate (β = −0·010, 95% CI: −0.017, −0.002, *p* = 0·013) but positively with fat (β = 0.022, 95% CI: 0.001, 0.043, *p* = 0.040) and protein (β = 0·023, 95% CI: 0.003, 0.043, *p* = 0·023).

**Table 3:** Impact of macronutrient variables on lipoprotein particles.

Lipoprotein particles	Mean ± SD		CHO	*P*-value	Fat	*P-*value	Protein	*P*-value
			β (95%CI)		β (95%CI)		β (95%CI)	
***Subclass concentrations***								
Total VLDL (nmol/L)	42·5 ± 17·6	Model 1	0·034 (0·010, 0·058)	0·006	0·038 (−0·033, 0·109)	0·299	0·07 (−0·005, 0·145)	0·067
		Model 2	0·009 (−0·037, 0·055)	0·703	−0·009 (−0·133, 0·115)	0·890	0·027 (−0·09, 0·145)	0·650
*l-VLDL (nmol/L)*	4·6 ± 3·8	Model 1	0·010 (0·005, 0·015)	<0·001	0·017 (0·002, 0·032)	0·023	0·017 (0·001, 0·033)	0·032
		Model 2	0·004 (−0·006, 0·013)	0·429	−0·003 (−0·029, 0·022)	0·785	−0·015 (−0·039, 0·009)	0·215
*m-VLDL (nmol/L)*	14·8 ± 9·4	Model 1	0·026 (0·013, 0·039)	<0·001	0·053 (0·014, 0·092)	0·008	0·076 (0·035, 0·117)	<0·001
		Model 2	−0·004 (−0·028, 0·021)	0·782	0·003 (−0·064, 0·070)	0·932	0·037 (−0·027, 0·100)	0·256
*s-VLDL (nmol/L)*	23·0 ± 11·7	Model 1	−0·002 (−0·019, 0·014)	0·782	−0·033 (−0·081, 0·016)	0·187	−0·023 (−0·075, 0·028)	0·376
		Model 2	0·009 (−0·024, 0·042)	0·600	−0·008 (−0·097, 0·081)	0·855	0·005 (−0·079, 0·089)	0·906
Total LDL (nmol/L)	1125 ± 347	Model 1	0·426 (−0·072, 0·924)	0·094	0·367 (−1·094, 1·828)	0·622	1·314 (−0·232, 2·86)	0·096
		Model 2	−0·523 (−1·36, 0·313)	0·219	0·979 (−1·286, 3·243)	0·396	1·978 (−0·167, 4·123)	0·071
*i- LDL (nmol/L)*	180 ± 88	Model 1	−0·011 (−0·138, 0·117)	0·869	−0·05 (−0·424, 0·323)	0·792	−0·121 (−0·517, 0·275)	0·550
		Model 2	−0·114 (−0·361, 0·133)	0·364	0·479 (−0·188, 1·145)	0·159	0·025 (−0·609, 0·659)	0·939
*l-LDL (nmol/L)*	465 ± 210	Model 1	−0·436 (−0·735, −0·137)	0·004	−0·578 (−1·458, 0·302)	0·197	0·152 (−0·782, 1·087)	0·749
		Model 2	−0·809 (−1·396, −0·221)	0·007	1·316 (−0·281, 2·914)	0·106	3·111 (1·614, 4·608)	<0·001
*s-LDL (nmol/L)*	484 ± 325	Model 1	0·873 (0·408, 1·337)	<0·001	0·996 (−0·378, 2·37)	0·155	1·282 (−0·174, 2·739)	0·084
		Model 2	0·399 (−0·391, 1·188)	0·322	−0·816 (−2·952, 1·321)	0·454	−1·157 (−3·185, 0·871)	0·263
Total HDL (µmol/L)	30·7 ± 4·6	Model 1	−0·002 (−0·009, 0·004)	0·462	0·015 (−0·004, 0·034)	0·132	0·017 (−0·003, 0·037)	0·095
		Model 2	−0·019 (−0·032, −0·006)	0·004	0·043 (0·008, 0·078)	0·015	0·046 (0·013, 0·079)	0·006
*l-HDL (µmol/L)*	6·3 ± 3·3	Model 1	−0·013 (−0·018, −0·008)	<0·001	−0·018 (−0·031, −0·004)	0·011	−0·021 (−0·036, −0·007)	0·004
		Model 2	−0·010 (−0·017, −0·002)	0·013	0·022 (0·001, 0·043)	0·040	0·023 (0·003, 0·043)	0·023
*m-HDL (µmol/L)*	9·4 ± 3·8	Model 1	0·000 (−0·006, 0·005)	0·962	0·008 (−0·008, 0·024)	0·311	0·007 (−0·009, 0·024)	0·383
		Model 2	−0·004 (−0·015, 0·007)	0·460	0·006 (−0·023, 0·035)	0·674	0·008 (−0·019, 0·036)	0·560
*s-HDL (µmol/L)*	*15·0* ± *4·7*	Model 1	0·011 (0·004, 0·017)	0·001	0·024 (0·005, 0·043)	0·013	0·031 (0·011, 0·051)	0·003
		Model 2	−0·005 (−0·017, 0·007)	0·411	0·015 (−0·018, 0·047)	0·371	0·015 (−0·015, 0·046)	0·332
***Particle size***								
VLDL (nm)	51·6 ± 8·5	Model 1	0·023 (0·011, 0·035)	<0·001	0·042 (0·007, 0·077)	0·018	0·041 (0·004, 0·078)	0·028
		Model 2	0·011 (−0·011, 0·034)	0·333	−0·026 (−0·086, 0·035)	0·406	−0·042 (−0·099, 0·016)	0·155
LDL (nm)	21·1 ± 0·6	Model 1	−0·002 (−0·003, −0·001)	<0·001	−0·003 (−0·005, 0·000)	0·037	−0·002 (−0·005, 0·000)	0·102
		Model 2	−0·001 (−0·003, 0·000)	0·111	0·002 (−0·002, 0·007)	0·303	0·005 (0·001, 0·009)	0·019
HDL (nm)	9·3 ± 0·5	Model 1	−0·002 (−0·003, −0·001)	<0·001	−0·003 (−0·005, −0·001)	0·004	−0·004 (−0·007, −0·002)	<0·001
		Model 2	−0·001 (−0·002, 0·000)	0·227	0·002 (−0·001, 0·005)	0·264	0·001 (−0·002, 0·004)	0·657

Abbreviations: BMI = body mass index; CHO = carbohydrate; GLM = general linear model; HDL = high density lipoprotein; i-LDL = intermediate-low density lipoprotein; LDL = low density lipoprotein; l-HDL = large-high density lipoprotein; l-LDL = large-low density lipoprotein; l-VLDL = large-very low density lipoprotein; m-VLDL = medium-very low density lipoprotein; m-HDL = medium-high density lipoprotein; PAL = physical activity level; s-HDL = small-high density lipoprotein; s-LDL = small-low density lipoprotein; s-VLDL = small-very low density lipoprotein; VLDL = very-low density lipoprotein.

Note- Carbohydrate, fat and protein intakes were used as dependent variable with all lipoprotein subclasses and particle sizes as independent variables.

Model 1: Multivariate analysis (GLM), unadjusted.

Model 2: Multivariate analysis (GLM), adjusted for age, BMI, PAL, sex and total energy intake. Also refer Supplementary Table 3: Lipoprotein concentrations and particle sizes as per quartiles of total energy and macronutrient intakes.

Only LDL particle size was associated with protein (β = 0·005, 95% CI: 0.001, 0.009, *p* = 0·019).

### Associations with fat-carbohydrate combinations

**Figure 2:**
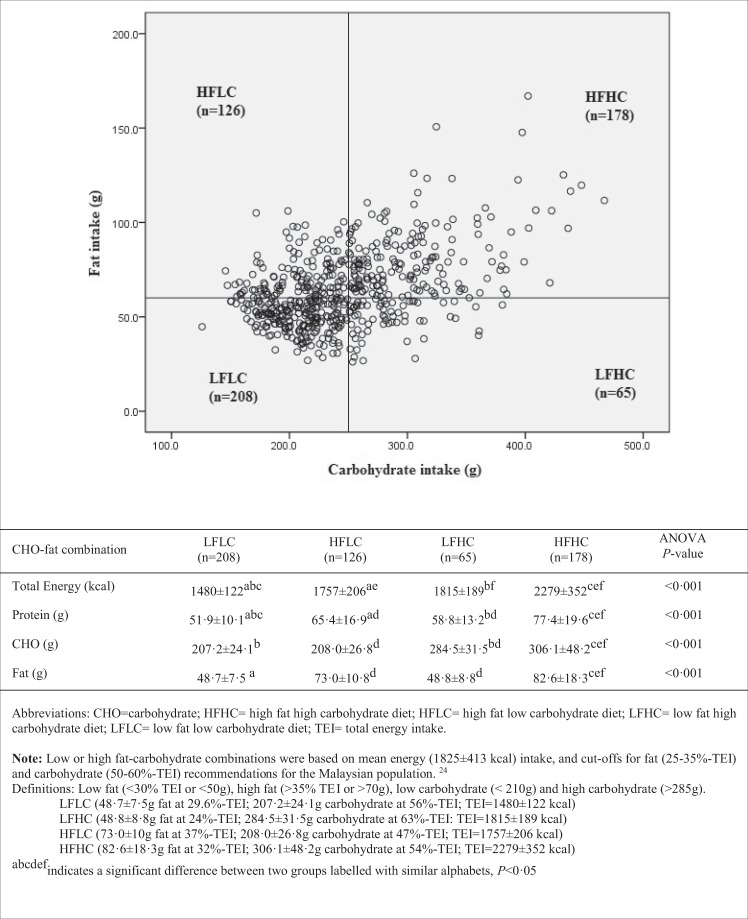
Fat-carbohydrate intake patterns of MLS population groupings as per 55% carbohydrate-TEI and 30% fat-TEI limits.

We further examined the associations of fat-carbohydrate combinations. Fat-carbohydrate intake groupings, based on 250 g carbohydrate and 60 g fat cut-off limits, yielded low-fat, low-carbohydrate (LFLC), low-fat, high-carbohydrate (LFHC), high-fat, low-carbohydrate (HFLC), high-fat, high-carbohydrate (HFHC) groups^24^. Figure 2 indicates fat-carbohydrate content distributions for these diet permutations. Subjects’ cardio-biomarker profile based on these four combinations are presented in Table 4.

**Table 4:** Cardio-biomarker profile of fat-carbohydrate combination diets.

Fat-carbohydrate combinations	LFLC n = 208	HFLC n = 126	LFHC n = 65	HFHC n = 178	GLM *(P*-value)	
					*Model 1*	*Model 2*
***Cardio-biomarkers***						
TC (mmol/L)	5.20 ± 0.94	5.10 ± 0.89	5.09 ± 0.90	5.06 ± 0.87	0.464	0.232
HDL-C (mmol/L)	1.53 ± 0.40^a^	1.51 ± 0.41	1.43 ± 0.39	1.39 ± 0.37^a^	0.010	0.600
TG (mmol/L)	1.09 ± 0.50	1.12 ± 0.55	1.36 ± 0.80	1.24 ± 0.66	0.078	0.556
LDL-C (mmol/L)	3.16 ± 0.90	3.08 ± 0.82	3.03 ± 0.82	3.10 ± 0.84	0.777	0.160
VLDL-C (mmol/L)	0.50 ± 0.23	0.51 ± 0.25	0.63 ± 0.37	0.57 ± 0.30	0.078	0.556
TC:HDL-C	3.61 ± 1.12	3.63 ± 1.20	3.82 ± 1.24	3.88 ± 1.20	0.777	0.743
LDL-C:HDL-C	2.23 ± 0.95	2.24 ± 0.99	2.29 ± 0.86	2.41 ± 0.97	0.365	0.534
Glucose (mmol/L)	5.11 ± 1.01	5.30 ± 1.23	4.96 ± 0.56	5.35 ± 1.16	0.077	0.015
Insulin (uu/mL)	5.65 ± 3.74^a^	5.87 ± 3.93^b^	5.82 ± 3.87^c^	7.62 ± 6.23^a,b,c^	<0.001	<0.001
HOMA2-IR	0.77 ± 0.47^a^	0.79 ± 0.49^b^	0.79 ± 0.47^c^	1.03 ± 0.79^a,b,c^	<0.001	<0.001
SBP (mmHg)	121 ± 16^ab^	121 ± 15	128 ± 17^a^	126 ± 16^b^	0.002	0.190
DBP (mmHg)	74 ± 10^a^	74 ± 10	78 ± 10	77 ± 12^a^	0.009	0.255
Waist circumference (cm)	81·6 ± 12·0	83·3 ± 11·2	84·8 ± 11·3	87·7 ± 15·8	<0.001	0.095
hsCRP (mg/L)	3·11 ± 5·01	2·57 ± 3·85	3·56 ± 5·36	3·03 ± 4·58	0·550	0·535
***Lipoprotein subclass concentrations***						
Total VLDL (nmol/L)	40.8 ± 16.2	42.4 ± 17.8	44.1 ± 19.6	44.0 ± 18.0	0.567	0.949
l-VLDL (nmol/L)	4.0 ± 3.2	4.6 ± 3.8	5.3 ± 4.9	5.1 ± 4.0	0.096	0.899
m-VLDL (nmol/L)	12.7 ± 8.1^a,b^	15.1 ± 9.8	17.3 ± 10.9^a^	16.0 ± 9.7^b^	0.004	0.140
s-VLDL (nmol/L)	23.9 ± 11.8	22.6 ± 11.4	21.6 ± 12.6	22.9 ± 11.6	0.374	0.354
Total LDL (nmol/L)	1106 ± 325	1131 ± 370	1147 ± 383	1136 ± 343	0.823	0.659
i-LDL (nmol/L)	180 ± 88	190 ± 92	181 ± 92	172 ± 84	0.607	0.359
l-LDL (nmol/L)	494 ± 211^a^	477 ± 225	425 ± 206	436 ± 194^a^	0.028	0.047
s-LDL (nmol/L)	433 ± 298^a,b^	470 ± 350	547 ± 314^a^	529 ± 332^b^	0.035	0.561
Total HDL (µmol/L)	30.3 ± 4.9	31.1 ± 4.6	31.0 ± 5.2	30.6 ± 4.0	0.471	0.115
l-HDL (µmol/L)	6.8 ± 3.3^a^	6.7 ± 3.4^b^	5.8 ± 2.9	5.5 ± 3.1^a,b^	0.001	0.301
m-HDL (µmol/L)	9.3 ± 3.8	9.5 ± 3.7	9.6 ± 4.0	9.4 ± 3.7	0.964	0.983
s-HDL (µmol/L)	14.2 ± 5.0^a^	14.9 ± 4.9	15.8 ± 4.0	15.7 ± 4.5^a^	0.020	0.372
***Lipoprotein particle size***						
VLDL (nm)	50.1 ± 8.2^a^	51.7 ± 7.7	52.8 ± 9.4	52.8 ± 8.8^a^	0.028	0.588
LDL (nm)	21.2 ± 0.6^a^	21.1 ± 0.6^b^	20.9 ± 0.6	20.9 ± 0.6^a,b^	0.001	0.074
HDL (nm)	9.4 ± 0.5^a^	9.3 ± 0.5	9.2 ± 0.5	9.2 ± 0.5^a^	0.002	0.826

Abbreviations: BMI = body mass index; BPdiastolic = diastolic blood pressure; BPsystolic = systolic blood pressure; GLM = general linear model; HDL = high density lipoprotein; HDL-C = high density lipoprotein-cholesterol; HOMA2- IR = homeostatic model assessment of insulin resistance; i-LDL = intermediate-low density lipoprotein; LDL = low density lipoprotein; LDL-C = low density lipoprotein-cholesterol; l-HDL = large-high density lipoprotein; l-LDL = large-low density lipoprotein; l-VLDL = large-very low density lipoprotein; m-VLDL = medium-very low density lipoprotein; m-HDL = medium-high density lipoprotein; PAL = physical activity level; s-HDL = small-high density lipoprotein; s- LDL = small-low density lipoprotein; s-VLDL = small-very low density lipoprotein; TC = total cholesterol; TG = triglyceride; VLDL = very low density lipoprotein; VLDL-C = very low density lipoprotein-cholesterol.

Note-

Model 1: Multivariate analysis, unadjusted.

Model 2: Multivariate analysis, GLM, adjusted for age, BMI, PAL and sex.

^a,b,c,d,e,f^ indicates a significant difference between two groups labelled with similar alphabets, *P* < 0.05. Also refer Supplementary Table 4: Multivariate analyses (GLM) for association between macronutrient intake and cardio-biomarkers with unadjusted and adjusted models using different levels of confounding factors.

Plasma glucose was not significantly different between the 4 fat-carbohydrate groups although the trend was significant in the adjusted model (*P* = 0.015), attributed to higher HFLC and HFHC group values. Plasma insulin registered significant elevations in the HFHC group (7.62 ± 6.23 uu/mL, *P* < 0·001) compared to the other three groups (LFLC = 5.65 ± 3.74, HFLC = 5.87 ± 3.93 and HFLC = 5.82 ± 3.87 uu/mL), which persisted strongly (*P* < 0·001) in both our analytical models. HOMA2-IR results were in tandem with insulin trends.

Significantly lower HDL-C concentrations in the HFHC *vs*. LFLC group (*P* = 0·010) were observed but this difference became non-significant with adjusted analyses. However, LFHC and HFHC groups consuming carbohydrate-rich diets had significantly higher systolic (128 ± 17 and 126 ± 16 mmHg respectively, *P* = 0·002) and diastolic (78 ± 10 and 77 ± 12 mmHg respectively, *P* = 0·009) BP although these effects were attenuated after adjustments for co-variates.

In the unadjusted model, significant differences (all *P* < 0.05) in lipoprotein particle size (VLDL, LDL, and HDL), and concentrations (medium-VLDL, large-LDL, small-LDL, large-HDL and small-HDL) were observed in the HFHC *vs*. LFLC groups.

LFLC compared to the carbohydrate-rich LFHC and HFHC was associated with less atherogenic lipoprotein particle patterns, as indicated by significantly lowest concentrations in medium-VLDL concentrations (LFLC, 12.7 ± 8.1 < HFHC, 16.0 ± 9.7 nmol/L < LFHC, 17.3 ± 10.9, *P* = 0.004) and small-LDL (LFLC, 433 ± 298 < HFHC, 529 ± 332 < LFHC, 547 ± 314 nmol/L, *P* = 0.035). In particular, LFLC had significantly (*P* = 0.035) higher large-LDL concentrations compared to HFHC ((494 ± 211 *vs*. 436 ± 194 nmol/L). When tested using the adjusted Model 2, these differences became non-significant except for the observed higher large-LDL numbers in the HFHC *vs.* LFLC diets (*P* = 0·047).

### Risks for insulin resistance and atherogenic lipoproteins

We examined the odds ratios (OR) for the fat-carbohydrate groups potentially impacting atherogenicity and insulin resistance, with the LFLC ascribed an OR of 1·0 (Table 5). In the unadjusted model, risk associated with HFHC group was significant and highest for TC:HDL-C > 4·5 mmol/L (OR = 1·68, 95%CI: 1·05, 2·69), HOMA2-IR > 1·7 (OR = 3·31, 95%CI: 1·64, 6·69), large-LDL particles <450 nmol/L (OR = 1·67, 95%CI: 1·11, 2·51) and small-LDL particles >600 nmol/L (OR = 1·71, 95%CI: 1·11, 2·61). After adjusting for age, BMI, PAL and sex, the odds of having HOMA2-IR > 1.7 in the HFHC group was 2.43 (95%CI: 1·03, 5·72) times more compared to the LFLC diet group and odds of having large-LDL < 450 nmol/L in the LFHC group was 1.91 (95%CI: 1·06, 3·44) more compared to the LFLC diet group. In addition, the comparative OR between LFLC and LFHC emerged significant for large-LDL particles (OR = 1·91, 95%CI: 1·06, 3·44).

**Table 5:** Odds ratios for diet groups as per risk categories.

	LFLC	HFLC	LFHC	HFHC
TC:HDL-C > 4.5 mmol/L				
Model 1	1	1.06 (0.61, 1.84)	1.33 (0.69, 2.57)	1.68 (1.05, 2.69)^#^
Model2	1	0.85 (0.45, 1.58)	0.98 (0.47, 2.02)	0.95 (0.53, 1.69)
HOMA2-IR > 1.7				
Model 1	1	1.11 (0.44, 2.79)	0.52 (0.11, 2.38)	3.31 (1.64, 6.69)^#^
Model2	1	0.93 (0.34, 2.54)	0.38 (0.08, 1.92)	2.43 (1.03, 5.72)^#^
Large-LDL < 450 nmol/L				
Model 1	1	1.45 (0.92, 2.27)	1.82 (1.02, 3.22)^#^	1.67 (1.11, 2.51)^#^
Model 2	1	1.30 (0.82, 2.07)	1.91 (1.06, 3.44)^#^	1.49 (0.95, 2.34)
Small-LDL > 600 nmol/L				
Model 1	1	1.07 (0.66, 1.74)	1.61 (0.90, 2.88)	1.71 (1.11, 2.61)^#^
Model 2	1	0.96 (0.56, 1.66)	1.23 (0.65, 2.33)	1.15 (0.69, 1.94)

Abbreviations: HDL-C = high density lipoprotein-cholesterol; HFHC = high fat high carbohydrate group; HFLC = high fat low carbohydrate group; HOMA2-IR = homeostatic model assessment of insulin resistance; LFHC = low fat high carbohydrate group; LFLC = low fat low carbohydrate group; large-LDL = large-low density lipoprotein particle; small-LDL = small- low density lipoprotein particle; TC = total cholesterol.

Note**-**

Model 1 – Logistic regression, unadjusted.

Model 2 – Logistic regression, adjusted for age, BMI, PAL and sex, not adjusted for total energy.

*Significantly different from LFLC group.

## Discussion

As many Asian economies stride toward developed status, population trends towards higher incidence of risks for vasculopathy including CVD, type 2-diabetes and MetS is also advancing rapidly^2–4^. This is a major public health concern and significant efforts are being directed at re-examining the changing dietary patterns in the region as primary disease causative agents^7,8^.

The on-going debate among experts on the role of total calories, fats especially, saturated fat, and excess carbohydrate consumption as drivers of global health, makes it supremely difficult to offer precise population-based dietary recommendations^11,25,26^. Furthermore, a recent systematic review and meta-analysis reveal national dietary guidelines in the United States and United Kingdom aimed at reducing CHD incidence through reduced total and saturated fat intake were not supported by evidence from randomized controlled trials^9,10,27^. In particular, recommendations for higher consumption of PUFA and lower consumption of saturated fats, as part of cardiovascular health guidelines, were suggested to be not evidence-based^9,10^. Recently this was challenged by Wang *et al*. 2016, who concluded based on two prospective cohort observations that intake of ω6-PUFA, especially linoleic acid, was inversely associated with mortality^28^. To improve population health, the food industry supportive of dietary guidelines has pitched low-fat foods as healthy choices, which have resulted in increased dietary exposure to carbohydrates^29^. Increased consumption of refined carbohydrates has been demonized as a dietary causative factor for diabetes and obesity^30^. In the United Kingdom, health guidelines are divided between conservative positions on fat restriction or focus on restricting carbohydrates^10,30^.

From this discourse emerges confusion about the extent to which dietary macronutrients contribute either singly or in some combination toward chronic disease burden. Throughout Asia, Western dietary recommendations have been instituted without adequate or reliable regional data. Further, it is acknowledged that global data on dietary fats and oils identify dramatic diversity across nations, hence obscuring the link to country-specific health data and clinical reality^31^. We therefore undertook the MLS study to address the role of macronutrient consumption that may be associated with various disease risk indicators and included additional lipoprotein particles assessment for more precise indicators of CVD risk in a relatively circumscribed if not discrete population. The reported prevalence of MetS in Malaysia is 27·5%, whereas we found prevalence of 16·3% in the MLS population; a lower trend which may be explained by our study’s exclusion criteria at recruitment of those previously diagnosed with diabetes, hypertension and hypercholesterolemia^32^. In addition association between the genetic risk score and Type 2 diabetes for Malays, Chinese and Indians in Malaysia was low (1.6, 1.7 and 1.0%, respectively) based on 62 identified single nucleotide polymorphisms for Type 2 diabetes^33^. Thus if the different ethnic groups have an equal chance of developing NCDs, then it is highly likely that diet is an important environmental factor contributing to the NCD burden in Malaysia.

In the MLS population, saturated fat consumption was relatively high (~14%-TEI) primarily due to the dominance of POL as the major dietary oil consumed. PUFA intake was <5%-TEI in this population which is consistent with global consumption trends^34^. We found there was no significant association between fat intake and most cardio-biomarkers, except insulin and HOMA2-IR scores. These however, were rendered insignificant in the adjusted model, while glucose became positively associated, and SBP was negatively associated. Instead, high carbohydrate intakes in the MLS population correlated to dyslipidemia, represented by higher triglycerides and decreased HDL-C coupled with increased small-LDL particles as well as characteristics of central obesity, insulin resistance and hypertension in the unadjusted model. However, effects prevailed only on HDL-C and insulin in the adjusted model. The impact of higher carbohydrate consumption in this population on lipid and diabetes associated risk factors is consistent with the view that the features of MetS relate to defective insulin action including inflammation and altered fatty acid partitioning^35^. Increased large-LDL particles which are considered beneficial in lowering atherogenic risk, correlated with higher protein intakes in the MLS population whereas higher carbohydrate intakes mediated an inverse relationship^16^. Large- HDL particle concentrations were negatively correlated with carbohydrate intake but positively correlated with protein and fat intakes.

We further characterized the dietary patterns of the MLS population into four fat-carbohydrate permutations; two low fat (LFLC, LFHC) and two high fat (HFLC, HFHC) combinations. The HFHC diet increased insulin resistance when compared to the other fat-carbohydrate combinations. This outcome supports assertions that high dietary total energy intake, perhaps driven by high carbohydrate and fat calorie intakes reduce insulin sensitivity, thus increasing risk of diabetes^35^. In the MLS population, this relationship remained consistent even after adjustment for covariates. The two low fat diets (LFLC, LFHC) did not result in the anticipated improved plasma lipid CHD-risk profiles compared to the high fat diets (HFLC, HFHC). Only HDL-C was significantly lower with the HFHC diet compared to the LFLC diet. The most interesting observation was that large LDLs previously suggested as less atherogenic were progressively increased by fat-carbohydrate diet permutations in the following order: LFLC > HFLC > LFHC > HFHC with the difference between LFLC and HFHC attaining significance^16^. Small putatively atherogenic lipoprotein particles moved in the opposite direction and were concomitantly reduced in the order: LFLC < HFLC < LFHC < HFHC. This suggests a potential detrimental modulatory role for excess carbohydrate consumption with respect to lipoprotein particles and atherogenicity.

A putative atherogenic quality of carbohydrates modulated through unfavorable impact on lipoprotein particle classes as observed from the MLS data, underscores emerging concerns regarding overconsumption of carbohydrates, at least in the studied population^13,29^. Clues for the behavior of lipoprotein subclasses can be drawn from clinical trials^36–38^. For example, a 12-week very-low carbohydrate diet significantly altered LDL particle distribution by increasing the concentration for large-LDL with a concomitant decrease in small-LDL and LDL particle size^36^. In contrast, a high carbohydrate but low fat diet caused reductions in large-LDL and an increment in small-LDL concentrations^37^. Another 12-week intervention study with a low carbohydrate but high fat diet, altered lipoprotein metabolism by favorably modifying the VLDL, LDL and HDL subclasses distribution and their size. Both large-LDL and large-HDL concentrations increased whereas small-LDL decreased^38^. Metabolically, higher total energy and carbohydrate intakes have been shown to increase hepatic TAG level, resulting in the formation of large TAG-enriched lipoprotein particles, for instance large-VLDL, that ultimately become small-LDL after a series of lipolysis and remodeling^39^. Low dietary carbohydrate intake on the other hand may result in low hepatic TAG levels which is suggested to stimulate secretion of small-VLDL, giving rise to large-LDL after lipolysis and decrease of small-LDL in circulation.  A high carbohydrate-low fat intake may result in lower large-LDL but higher small-LDL particle concentration in circulation was also observed in this study cohort.

These MLS data overall support the hypothesis that cardiometabolic health may benefit to a greater extent with restrictions on carbohydrate consumption rather than total fat^11,12,26^. Experimental reduction in dietary carbohydrates in human studies was shown to lead to improvements through reduced TAG and increased HDL-C, overall MetS and diabetes, even without weight loss or even in the presence of high saturated fat intake^36–38^. The basis for this may be explained using hypocaloric carbohydrate restricted (~14%-en) diets high in SFA which indicated that not only did SFA dose bear limited effect on plasma SFA but SFA was efficiently metabolized in the presence of low carbohydrate^36^.

Within a low carbohydrate environment (<210 g) with fat at either <30%-TEI or >35%-TEI and protein at approximately 15–20%-TEI, the low fat LFLC diet compared to the high fat HFLC diet did not result in any noteworthy advantages with respect to various cardiometabolic indicators assessed. This observation contrasts with various expert dietary recommendations focusing on fat and specifically saturated fat consumption. When high fat in the diet was coupled with high carbohydrate intake (HFHC), a number of the plasma CHD risk indicators were significantly altered and this signaled potential concerns. It thus appears that this fat-carbohydrate combination rather than fat intake alone should be actively monitored and the impact rigorously explored in future dietary intervention and/or population studies. High carbohydrate intakes are typical for most Asian populations and integral to sociocultural identities, with consumption of either white rice or wheat-products forming the basis of these diets^40^.

Based on the fat-carbohydrate combination diets, we calculated the odds ratio (OR) for potential impact on cardiometabolic risk in the MLS population. The HFHC diet combination more than doubled risk for insulin resistance defined by HOMA2-IR > 1.7 after adjustment for co-variates. It also showed propensity to be pro-atherogenic defined by higher OR for TC:HDL-C and small dense LDL particles, though these effects became non-significant in the adjusted model. Increased small, dense LDL particles have been associated with more than a three-fold increase in CVD risk in case control studies, and are further substantiated through its involvement in increasing carotid intima-media thickness, a measure of subclinical atherosclerosis^16,41^. The mechanism for increased atherogenic potential of small dense LDL is suggested to be greater propensity for transport into the subendothelial space, increased binding to arterial proteoglycans and susceptibility to oxidative modification^39^. Our data are suggestive that a high-fat, high-carbohydrate combination could play a significant role in modulating small dense LDL and hence overall CVD risk, as observed in the population we studied.

In contrast, we also observed that the LFHC diet in comparison to LFLC diet significantly raised OR for large buoyant LDL particles. Risk resulting from increases in both large and small LDL particle size on cardiovascular mortality is just emerging^41,42^. Our data underscores the potential of high carbohydrate diets even when accompanied by low fat (LFHC) or high fat (HFHC) combinations to influence overall LDL particle size.

Interestingly, we established that low grade inflammation, indicated by hsCRP measurements, was not linked to either individual macronutrient consumption levels or the fat-carbohydrate combinations in this population.

### Study limitations

We acknowledge that in our current dataset, the differences in both fat and carbohydrate intake between assigned dietary groups is small. The danger that such small differences may contribute potential biases in the outcomes reported has previously been outlined by Ioannidis^43^, who amplified that such estimated benefits could reflect cumulative research biases coupled with possible residual confounding and selective reporting of the data. Thus extrapolation of findings from this study may be limited by its cross-sectional nature, as well as its focus on a Malaysian diet and population. We also opted to extract food consumption details from self-reported dietary records rather than using a food frequency questionnaire as we needed exhaustive information on dietary fats and “foods away from home-based consumption”, which would otherwise be missed using these regular questionnaires. However, the data extracted from this cohort still yielded detailed information on dietary consumption patterns and cardiometabolic biomarkers for a MetS-prone population in a country where NCD-related mortality attributed to CVD has been reported to be high. A follow-up prospective study in this population to establish possible links between dietary patterns and risks assessment for CVD and diabetes is also deemed crucial.

## Conclusions

Our findings suggest a potential role for adjusting fat-carbohydrate dietary combinations in modulating insulin resistance and atherogenic risk in the Malaysian population that we studied. High carbohydrate intake (>285 g) coupled with high fat (>70 g) consumption was associated with negative impacts on CVD risk, especially on dyslipidemia and hypertension. This finding is also in accord with emerging evidence highlighting a prominent role for carbohydrates in CVD risk as opposed to total or saturated fat alone. Our traditional understanding of dietary factors and accompanying dietary recommendations for CVD management may require reassessment in light of this and other emerging evidence.

## Supplementary information

Table 1–4
