# Supplementary material for: A Cross-Sectional Study on the Dietary Pattern Impact on Cardiovascular Disease Biomarkers in Malaysia
Source: Sci Rep. 2019 Sep 20;9:13666. doi: 10.1038/s41598-019-49911-6 (PMC6754378; doi:10.1038/s41598-019-49911-6)
Supplement: Supplementary file 1 — Table 1–4 [file 41598_2019_49911_MOESM1_ESM.pdf]

Supplementary Information

Title: A Cross-Sectional Study on the Dietary Pattern Impact on Cardiovascular Disease Biomarkers in Malaysia

Authors

Tilakavati Karupaiah[1,2], Khun-Aik Chuah[2], Karuthan Chinna[3], Peter Pressman[4], Roger A. Clemens[5], A. Wallace Hayes[6], Kalyana Sundram[7]*

*Corresponding Author

Author affiliations:
[1]Tilakavati Karupaiah, PhD, SRI Professor, School of BioSciences, Faculty of Health & Medical Sciences, Taylor's University, Malaysia and [2]Visiting Professor, Faculty of Health Sciences, National University of Malaysia, Malaysia.
email: tilly_karu@yahoo.co.uk ; tilakavati.karupaiah@taylors.edu.my

[2]Khun-Aik Chuah, PhD, Nutrition Program, School of Healthcare Sciences, Faculty of Health Sciences, National University of Malaysia, Malaysia;
email: cruise_chuah@hotmail.com

[3] Karuthan Chinna, PhD, Associate Professor, Julius Center, Department of Social and Preventive Medicine, Faculty of Medicine, University of Malaya, Malaysia;
email: karuthan@gmail.com

[4]Peter Pressman, MD, MS, FACN, VP Medical Operations, Polyscience Consulting & Director of Nutrition and Public Health, The Daedalus Foundation, USA;
email: peter pressman <drpressvm2@gmail.com>

[5]Roger Clemens, DrPH, FIFT, CFS, FASN, FACN, CNS, FIAFST, Adjunct Professor, Pharmacology & Pharmaceutical Sciences, USC School of Pharmacy, International Center for Regulatory Science, USA;
email: clemens@usc.edu

[6]A. Wallace Hayes, PhD, DABT, FATS, FIBiol, FACFE, FACN, Adjunct Professor, Center for Integrative Toxicology, Michigan State University, USA;
email: A. Wallace Hayes <awallacehayes@comcast.net>

[7]Kalyana Sundram, PhD, FASc, FNSM, CEO, Malaysian Palm Oil Council, Kelana Jaya, Selangor, Malaysia;
email: kalyana@mpoc.org.my

Table 1: Total energy and macronutrient intakes as per quartiles of total energy and macronutrient intakes

	Quartile of Total Energy Intake				Quartile of Carbohydrate Intake				Quartile of Fat Intake				Quartile of Protein Intake			
Dietary Intake	Q1	Q2	Q3	Q4	Q1	Q2	Q3	Q4	Q1	Q2	Q3	Q4	Q1	Q2	Q3	Q4
Total energy (kcal)	1404±66	1616±66	1901·3±92·2	2382±325	1482±160	1634±199	1866±222	2321±380	1505±185	1634±211	1862±261	2298±384	1506±181	1659±237	1878±263	2263±421
Protein (g)	48·3±8·6	56·2±9·5	66·5±12·0	83·1±20·4	54·9±12·8	58·3±15·9	63·5±15·8	77·3±21·2	50·8±10·8	56·5±11·1	64·3±13·0	82·4±20·6	44·0±4·7	55·3±2·5	66·2±3·4	89·0±15·9
CHO (g)	195·0±23·9	220·4±28·3	254·0±30·4	317·6±51·5	183·2±15·2	220·8±9·0	256·0±11·9	327·0±41·2	228·2±39·6	224·8±46·9	247·8±57·0	285·8±63·4	217·4±36·1	231·3±47·2	252·0±50·9	286·5±67·7
Fat (g)	47·4±9·2	56·2±10·1	68·3±12·2	86·3±19·9	58·0±13·1	57·1±15·8	64·9±17·5	78·2±23·9	43·0±6·1	55·9±3·2	67·8±4·0	91·5±15·1	50·7±12·2	56·7±11·8	66·8±14·0	84·2±21·2
%-TEI Protein	13·8±2·3	14·0±2·5	14·0±2·4	13·9±2·7	14·8±2·6	14·1±2·4	13·5±2·3	13·2±2·2	13·5±2·3	13·9±2·5	13·8±2·3	14·3±2·7	11·8±1·5	13·6±1·8	14·4±2·0	15·9±2·5
%-TEI CHO	55·7±6·6	54·5±6·4	53·5±6·3	53·5±6·5	49·9±5·4	54·8±6·2	55·5±6·2	57·0±6·0	60·5±5·0	54·6±4·8	52·6±5·3	49·4±5·3	57·8±6·2	55·5±5·4	53·4±5·7	50·4±6·3
%-TEI Fat	30·4±5·6	31·3±5·5	32·2±5·3	32·4±5·3	35·0±4·7	30·9±5·1	30·8±5·2	29·8±5·3	25·9±3·9	31·2±3·8	33·3±4·4	36·0±4·0	30·3±6·0	30·8±4·9	32·0±5·1	33·4±5·4

Abbreviations: %-TEI=percentage of total energy intake; CHO=carbohydrate.

Table 2: Cardio-biomarkers profile as per quartiles of total energy and macronutrient intakes

	Quartile of Total Energy Intake				Quartile of Carbohydrate Intake				Quartile of Fat Intake				Quartile of Protein Intake			
Parameters	Q1	Q2	Q3	Q4	Q1	Q2	Q3	Q4	Q1	Q2	Q3	Q4	Q1	Q2	Q3	Q4
TC (mmol/L)	5.09±0.85	5.26±1.00	5.02±0.86	5.11±0.89	5.12±0.87	5.08±0.86	5.15±0.91	5.13±0.99	5.15±0.96	5.16±0.88	5.07±0.94	5.10±0.83	5.18±0.93	5.08±0.81	5.07±0.99	5.15±0.87
HDL-C (mmol/L)	1.52±0.38	1.51±0.42	1.48±0.40	1.37±0.36	1.55±0.38	1.50±0.40	1.49±0.41	1.35±0.35	1.49±0.39	1.51±0.40	1.51±0.39	1.38±0.38	1.52±0.39	1.48±0.39	1.47±0.40	1.42±0.39
TG (mmol/L)	1.07±0.53	1.19±0.52	1.13±0.63	1.30±0.72	1.07±0.54	1.09±0.49	1.20±0.61	1.34±0.73	1.19±0.63	1.13±0.56	1.10±0.52	1.27±0.70	1.12±0.51	1.18±0.58	1.13±0.61	1.26±0.72
LDL-C (mmol/L)	3.07±0.78	3.20±0.95	3.01±0.82	3.15±0.86	3.08±0.79	3.08±0.84	3.11±0.87	3.17±0.93	3.11±0.90	3.13±0.83	3.06±0.87	3.13±0.83	3.15±0.87	3.06±0.80	3.08±0.92	3.15±0.83
VLDL–C (mmol/L)	0.49±0.25	0.55±0.24	0.52±0.29	0.60±0.33	0.49±0.25	0.50±0.23	0.55±0.28	0.61±0.34	0.55±0.29	0.52±0.26	0.50±0.24	0.59±0.32	0.52±0.24	0.54±0.27	0.52±0.28	0.58±0.33
TC:HDL-C	3.55±1.07	3.74±120	3.63±1.20	3.97±1.21	3.51±1.06	3.62±1.15	3.71±1.18	4.04±1.27	3.67±1.13	3.66±1.16	3.59±1.15	3.97±1.26	3.62±1.08	3.70±1.24	3.68±1.22	3.89±1.18
LDL-C:HDL-C	2.17±0.87	2.33±1.03	2.22±0.92	2.47±0.97	2.15±0.86	2.25±0.97	2.28±0.96	2.51±0.99	2.25±0.92	2.25±0.92	2.21±0.95	2.47±1.01	2.23±0.90	2.27±1.00	2.27±0.98	2.41±0.93
TG:HDL-C	0.81±0.61	0.89±0.53	0.90±0.85	1.10±0.84	0.79±0.57	0.82±0.52	0.94±0.71	1.15±0.98	0.92±0.69	0.88±0.79	0.82±0.55	1.08±0.83	0.84±0.56	0.92±0.66	0.90±0.84	1.04±0.81
Glucose (mmol/L)	5.18±1.18	5.10±0.85	5.23±1.18	5.33±1.07	5.16±0.95	5.19±1.08	5.23±1.41	5.26±0.79	5.11±0.99	5.07±0.86	5.31±1.31	5.34±1.11	5.13±1.09	5.28±1.38	5.16±0.94	5.28±0.85
Insulin (uu/mL)	5.67±3.88	5.82±3.77	5.89±4.16	7.93±6.45	5.86±3.98	5.70±3.71	5.71±4.42	8.06±6.20	5.93±3.91	5.48±3.79	5.87±3.78	8.03±6.60	5.41±3.29	6.33±4.72	6.25±4.86	7.33±5.75
HOMA2-IR	0.78±0.50	0.79±0.47	0.80±0.53	1.06±0.82	0.80±0.51	0.77±0.46	0.79±0.57	1.07±0.78	0.80±0.49	0.74±0.47	0.81±0.48	1.08±0.84	0.74±0.41	0.87±0.60	0.84±0.61	0.98±0.74
$BP_{systolic}$ (mmHg)	119±14	122±16	125±15	126±18	120±14	121±15	125±16	128±17	122±16	123±16	123±16	125±16	119±14	123±17	125±17	125±16
$BP_{diastolic}$ (mmHg)	73±9	74±11	76±11	78±12	73±9	74±10	76±11	78±12	75±10	74±10	75±11	77±12	73±9	75±10	76±12	77±11
WC (cm)	81.8±11.7	83.1±12.8	83.7±12.6	88.3±15.0	81.4±11.5	82.9±11.9	83.7±14.0	88.9±14.4	84.1±12.1	81.3±11.5	84.9±14.2	86.6±14.6	83.6±13.6	83.3±14.0	83.1±11.4	86.9±13.7
hsCRP (mg/L)	3·29±5·05	3·18±5·60	2·61±3·15	3·00±4·60	2·60±3·75	3·13±4·80	3·26±5·98	3·07±3·87	3·20±4·56	3·53±6·21	2·71±3·75	2·63±3·78	3·12±4·98	3·40±5·55	3·15±4·86	2·40±2·89

Abbreviations: CHO=carbohydrate; $BP_{diastolic}$=diastolic blood pressure; $BP_{systolic}$=systolic blood pressure; HDL-C= high density lipoprotein cholesterol; HOMA-IR= Homeostatic model assessment of insulin resistance; LDL-C= low density lipoprotein cholesterol; TC= total cholesterol; TG=triglycerides; VLDL-C=very low density lipoprotein cholesterol; WC=waist circumference.

Table 3: Lipoprotein concentrations and particle sizes as per quartiles of total energy and macronutrient intakes

	Quartiles of total				Quartiles of CHO				Quartiles of Fat intake				Quartiles of			
	Q1	Q2	Q3	Q4	Q1	Q2	Q3	Q4	Q1	Q2	Q3	Q4	Q1	Q2	Q3	Q4
Lipoprotein particle subclass concentrations																
Total VLDL (nmol/L)	39·3±15·0	43·8±17·9	42·1±19·	44·7±17·4	39·7±15·3	41·8±18·	43·5±18·5	44·9±17·9	41·6±16·	41·8±17·9	42·1±17·	44·5±17·	40·6±15·2	43·0±18·8	41·5±17·	44·9±18·2
l-VLDL (nmol/L)	3·9±3·3	4·7±3·5	4·5±4·1	5·4±4·1	4·1±3·5	4·2±3·4	4·5±3·7	5·6±4·5	4·4±3·8	4·3±3·6	4·4±3·4	5·4±4·4	4·3±3·4	4·5±3·7	4·4±3·8	5·2±4·3
m-VLDL (nmol/L)	12·6±7·7	15·0±8·9	14·6±10·	16·8±10·3	13·3±7·9	13·5±9·4	14·8±9·7	17·4±10·1	14·1±9·5	13·7±8·6	14·4±9·2	16·8±10·	13·5±8·0	14·6±9·3	13·6±9·5	17·4±10·4
s-VLDL (nmol/L)	22·7±11·3	24·1±12·8	22·9±11·	22·4±11·5	22·1±11·1	24·0±11·	24·1±12·0	21·9±11·9	23·1±12·	23·6±12·0	23·3±11·	22·2±10·	22·8±11·7	23·8±12·4	23·4±11·	22·2±11·8
Total LDL (nmol/L)	1079±304	1149±355	1108±355	1165±367	1098±337	1105±325	1110±336	1189±383	1109±347	1125±329	1098±359	1168±352	1086±307	1131±349	1118±357	1167±371
i-LDL (nmol/L)	182±91	191±86	167±85	180±89	185±89	175±82	180±91	181±91	185±93	176±87	182±90	177±83	193±91	176±80	173±96	178±83
l-LDL (nmol/L)	478±209	478±233	462±202	441±191	488±228	480±195	464±214	426±197	461±215	480±208	477±227	441±186	446±216	483±208	467±191	464±223
s-LDL (nmol/L)	427±301	479±328	483±328	545±335	434±318	447±299	470±336	584±327	465±316	473±300	445±331	551±344	450±308	479±327	481±327	526±335
Total HDL (µmol/L)	30·5±5·2	30·7±4·9	30·6±4·2	30·9±4·1	31·2±4·8	30·0±4·5	30·8±4·8	30·7±4·2	30·0±4·6	31·0±5·3	31·0±4·5	30·7±3·9	30·2±4·3	30·9±5·4	30·5±4·6	31·1±4·0
l-HDL (µmol/L)	7·1±3·1	6·5±3·3	6·3±3·3	5·3±3·1	7·2±3·1	6·5±3·5	6·4±3·2	5·0±2·9	6·6±3·1	6·4±3·2	6·7±3·3	5·4±3·2	6·8±3·2	6·4±3·2	6·3±3·2	5·6±3·3
m-HDL (µmol/L)	9·3±3·7	9·6±4·1	9·2±3·4	9·7±3·8	9·4±3·9	9·6±3·6	9·3±4·0	9·5±3·6	9·0±3·3	9·7±4·3	9·6±4·0	9·5±3·5	9·2±3·4	9·6±4·1	9·5±3·7	9·5±3·9
s-HDL (µmol/L)	14·2±5·2	14·7±4·8	15·2±4·3	15·9±4·5	14·7±5·5	14·0±4·6	15·2±4·3	16·2±4·3	14·5±4·5	15·0±5·2	14·7±4·9	15·8±4·3	14·3±4·6	15·0±5·2	14·9±4·6	16·0±4·6
Lipoprotein particle size																
VLDL (nm)	50·2±8·6	51·4±7·8	51·2±8·4	53·6±8·9	50·4±8·2	50·7±7·7	51·3±8·7	54·0±9·0	51·2±9·2	50·7±7·6	51·3±8·0	53·3±8·9	51·1±8·6	51·2±8·2	51·1±8·1	53·1±8·9
LDL (nm)	21·2±0·7	21·1±0·6	21·0±0·6	21·0±0·6	21·2±0·6	21·1±0·6	21·1±0·7	20·9±0·6	21·1±0·7	21·1±0·6	21·1±0·6	20·9±0·6	21·1±0·7	21·1±0·6	21·0±0·6	21·0±0·6
HDL (nm)	9·4±0·5	9·3±0·5	9·3±0·5	9·1±0·5	9·4±0·5	9·3±0·6	9·3±0·5	9·1±0·5	9·4±0·5	9·3±0·5	9·3±0·5	9·2±0·5	9·4±0·5	9·3±0·5	9·3±0·5	9·2±0·5

Abbreviations: BMI= body mass index; $BP_{diastolic}$=diastolic blood pressure; $BP_{systolic}$=systolic blood pressure; CHO=carbohydrate; GLM=general linear model; HDL=high density lipoprotein; HDL-C= high density lipoprotein cholesterol; HOMA-IR= Homeostatic model assessment of insulin resistance; i-LDL=intermediate low density lipoprotein; LDL=low density lipoprotein; l-HDL=large high density lipoprotein; l-LDL=large low density lipoprotein; l-VLDL=large very low density lipoprotein; LDL-C= low density lipoprotein cholesterol; m-VLDL=medium very low density lipoprotein; m-HDL=medium high density lipoprotein; PAL=physical activity level; s-HDL=small high density lipoprotein; s- LDL=small low density lipoprotein; s-VLDL=small very low density lipoprotein; TC= total cholesterol; TG=triglycerides; VLDL=very low density lipoprotein; VLDL-C=very low density lipoprotein cholesterol; WC=waist circumference.

Table 4: Multivariate analyses (GLM) for association between macronutrient intake and cardio-biomarkers with unadjusted and adjusted models using different level of confounding factors

Dependent variables	CHO					Fat					Protein				
	Model 1	Model 2	Model 3	Model 4	Model 5	Model 1	Model 2	Model 3	Model 4	Model 5	Model 1	Model 2	Model 3	Model 4	Model 5
	P-value	*P*-value	*P*-value	*P*-value	*P*-value	*P*-value	*P*-value	*P*-value	*P*-value	*P*-value	*P*-value	*P*-value	*P*-value	*P*-value	*P*-value
TC	0.984	0.517	0.510	0.252	0.250	0.460	0.433	0.640	0.331	0.309	0.770	0.347	0.232	0.042	0.050
TG	<0.001	0.004	<0.001	0.105	0.099	0.262	0.004	0.079	0.108	0.141	0.095	0.152	0.043	0.447	0.267
VLDL-C	<0.001	0.004	<0.001	0.105	0.099	0.262	0.004	0.079	0.108	0.141	0.095	0.152	0.043	0.447	0.267
LDL-C	0.461	0.411	0.867	0.424	0.415	0.855	0.378	0.535	0.483	0.416	0.486	0.508	0.217	0.102	0.144
HDL-C	<0.001	0.018	<0.001	0.036	0.028	0.036	0.031	0.125	0.058	0.082	0.042	0.079	0.171	0.085	0.020
TC:HDL-C	<0.001	0.031	0.005	0.350	0.331	0.122	0.046	0.065	0.438	0.586	0.063	0.297	0.060	0.677	0.342
LDL-C:HDL-C	0.002	0.091	0.044	0.630	0.626	0.186	0.125	0.127	0.738	0.923	0.110	0.463	0.121	0.918	0.567
TG:HDL-C	<0.001	0.006	<0.001	0.072	0.064	0.094	0.008	0.049	0.094	0.128	0.048	0.113	0.042	0.267	0.121
GLUCOSE	0.331	0.507	0.945	0.039	0.038	0.044	0.181	0.008	0.005	0.004	0.597	0.271	0.514	0.557	0.470
Insulin	<0.001	0.931	0.002	0.599	0.598	<0.001	0.574	<0.001	0.290	0.282	0.001	0.339	0.014	0.299	0.284
HOMA2-IR	<0.001	0.903	0.001	0.531	0.530	<0.001	0.481	<0.001	0.198	0.189	0.001	0.260	0.015	0.224	0.207
$BP_{systolic}$	<0.001	<0.001	<0.001	0.077	0.074	0.187	<0.001	0.011	0.022	0.029	0.005	0.402	<0.001	0.661	0.866
$BP_{diastolic}$	<0.001	0.002	<0.001	0.084	0.083	0.099	0.001	0.040	0.053	0.066	0.008	0.358	0.004	0.930	0.761
WC	<0.001	0.004	<0.001	0.013	0.008	0.006	0.031	0.002	0.199	0.284	0.022	0.021	0.070	0.003	<0.001
hsCRP (mg/L)	0.707	0.068	0.577	0.169	0.170	0.206	0.190	0.087	0.422	0.402	0.191	0.192	0.051	0.224	0.257
Total VLDL	0.006	0.110	0.012	0.722	0.703	0.299	0.128	0.036	0.798	0.890	0.067	0.939	0.010	0.455	0.650
l-VLDL	<0.001	0.121	0.001	0.455	0.429	0.023	0.206	0.011	0.682	0.785	0.032	0.265	0.034	0.404	0.215
m-VLDL	<0.001	0.369	0.001	0.772	0.782	0.008	0.225	0.001	0.971	0.932	<0.001	0.454	<0.001	0.157	0.256
s-VLDL	0.782	0.252	0.931	0.604	0.600	0.187	0.382	0.647	0.827	0.855	0.376	0.883	0.849	0.835	0.906
Total LDL	0.094	0.394	0.568	0.221	0.219	0.622	0.199	0.123	0.475	0.396	0.096	0.391	0.018	0.038	0.071
i- LDL	0.869	0.744	0.533	0.362	0.364	0.792	0.916	0.548	0.172	0.159	0.550	0.633	0.969	0.859	0.939
l-LDL	0.004	0.055	0.005	0.007	0.007	0.197	0.340	0.621	0.099	0.106	0.749	0.001	0.281	<0.001	<0.001
s-LDL	<0.001	0.040	0.005	0.344	0.322	0.155	0.044	0.073	0.385	0.454	0.084	0.264	0.089	0.459	0.263
Total HDL	0.462	0.023	0.469	0.004	0.004	0.132	0.048	0.039	0.012	0.015	0.095	0.036	0.031	0.014	0.006
l-HDL	<0.001	0.006	<0.001	0.026	0.013	0.011	0.008	0.014	0.033	0.040	0.004	0.127	0.010	0.157	0.023
m-HDL	0.962	0.255	0.897	0.463	0.460	0.311	0.370	0.465	0.654	0.674	0.383	0.534	0.440	0.609	0.560
s-HDL	0.001	0.547	0.010	0.408	0.411	0.013	0.511	0.001	0.435	0.371	0.003	0.610	0.001	0.225	0.332
VLDL particle size (nm)	<0.001	0.195	0.001	0.346	0.333	0.018	0.197	0.029	0.357	0.406	0.028	0.227	0.066	0.251	0.155
LDL particle size (nm)	<0.001	0.026	<0.001	0.121	0.111	0.037	0.063	0.025	0.261	0.303	0.102	0.030	0.138	0.043	0.019
HDL particle size (nm)	<0.001	0.044	<0.001	0.291	0.227	0.004	0.027	0.001	0.202	0.264	<0.001	0.850	<0.001	0.793	0.657

Abbreviations: BMI= body mass index; $BP_{diastolic}$=diastolic blood pressure; $BP_{systolic}$=systolic blood pressure; CHO=carbohydrate; GLM=general linear model; HDL=high density lipoprotein; HDL-C= high density lipoprotein cholesterol; HOMA2-IR= Homeostatic model assessment of insulin resistance; i-LDL=intermediate low density lipoprotein; LDL=low density lipoprotein; l-HDL=large high density lipoprotein; l-LDL=large low density lipoprotein; l-

VLDL=largeverylowdensitylipoprotein;LDL-C=lowdensitylipoproteincholesterol;m-VLDL=mediumverylowdensitylipoprotein;m-HDL=mediumhigh densitylipoprotein;PAL=physicalactivitylevel;s-HDL=smallhighdensitylipoprotein;s-LDL=smalllowdensitylipoprotein;s-VLDL=smallverylowdensity lipoprotein; TC= total cholesterol; TG=triglycerides; VLDL=very low density lipoprotein; VLDL-C=very low density lipoprotein cholesterol; WC=waist circumference.

Note-

Model 1: Unadjusted
Model 2: Adjusted for total energy intake only
Model 3: Adjusted for age, BMI & PAL only
Model 4: Adjusted for age, BMI, PAL & total energy intake only
Model 5: Adjusted for age, BMI, PAL, total energy intake and sex only
